# Supplementary material for: Barriers, enablers and outcomes reported by parents engaged with the special educational needs system in England: A qualitative study
Source: PLoS One. 2025 Nov 7;20(11):e0335606. doi: 10.1371/journal.pone.0335606 (PMC12594389; doi:10.1371/journal.pone.0335606)
Supplement: S1 File — (DOCX) [file pone.0335606.s001.docx]

## Supplementary file: Barriers, enablers and outcomes reported by parents/carers engaged with the special educational needs system in England: A qualitative study

## 1.0 Additional details about participant recruitment and attrition

After emailing parents/carers to gauge initial interest in taking part in this study 32/47 (68%) confirmed (the remainder did not reply or their email addresses were no longer working). There were a range of reasons why 10/32 dropped out of the study, including illness and needing to reschedule but being unable to find a convenient time. Two interviews were held in person at the respondents’ homes; 20 were online.

## 2.0 Semi-structured interview guide

**Parent and Carer Interview Schedule**

| **Introduction**   - Thank you for agreeing to talk to me today. - You helped with our HOPE survey because you have a child with special educational needs - Similar to the survey this interview will be based on your experiences with your youngest child with SEN - I am really interested in finding out more about your child’s experiences relating to the help and support they have received for their learning and development - Specifically what you think has worked well for your child, and maybe less well, and how it could be better… - And because this changes over time, we’re going to be using a timeline - This is a way of collecting information but also to help us talk today about your youngest child with SEN experiences of school and the support they’ve received so far - Our conversation will be private and what you say will be kept confidential, unless I think you, or your child are at risk of harm. In these instances, I will be legally obliged to report this to someone else, however this will not happen without your awareness and involvement. - I would like you to feel comfortable knowing that anything that you share throughout the conversation will be anonymised, so you won’t be identifiable in any published work. - It will take around one hour to complete… - (In-person)The conversation will be recorded on this device (show Dictaphone) and only the research team will have access to the recording - (Online) The conversation will be recorded on Zoom and only the research team will have access to the recording - You can pause or stop the interview at any point without giving an explanation      - **So are you okay to start this timeline together?** - So let’s start by adding some dates to your timeline, middle is ‘Today’ (add date), Past= When was your child born? Future= Date in 5 years time? | ***KEY AREAS TO COVER***      ***Thanks***      ***Purpose of the interview***      ***Youngest child with SEN***      ***Explanation of process***      ***Ethics and safeguarding***      ***Recording*** |
| --- | --- |

| **Current provision: Today**   - I’d like to begin in the present and by asking what stage of education (name) is currently in   (Pre-school, Nursey, KS1, KS2, KS3, KS4, A-levels/college, higher education, in work)     - Would you like to choose a colour to start filling the timeline in with      - How is (name) currently getting on in school/education/work…      1. Can you tell me what’s going well? What they enjoy most…      1. How about what’s harder? What they don’t enjoy so much…      1. Could you tell me about the impact their SEN has on their learning?      1. What kind of support is (name) getting at the moment? 2. Is this working for them? 3. What’s going less well? 4. Is there anyone in particular who helps them?      1. Who decided that this was the right support for (name)? 2. Were you asked? 3. Who, when, how?      1. Do you think this is the help they need? 2. What else do you think would be useful? Why? 3. How is it being monitored? 4. ***Has this support had an impact on their health?***      1. Have you suggested any of the changes to anyone at school/in the health services/local authority? | ***Address research questions:***     1. **What are the effects of SEN provision on the Health Outcomes of Children and Young People with SEN?**      1. **Is SEN provision fair and equitable across the country?**       ***Key Areas to cover***    ***What is current SEN intervention/support?***  ***Current SEN Intervention/support:***   - *What/Any others?* - *When/Timely?* - *Why/Does it match need?* - *Who/Services/ How is it decided?* - *What is working/enablers* - *How could it be better/barriers* - *Made a difference/How?*     ***Has this support had an impact on their health?*** |
| --- | --- |
| **The past**     - **Okay, so now I liked to talk to you about (name) experiences of (school and) learning when they were younger… We’ll move into the past on the timeline…**      - **Would you like to choose a different colour for the past or stay with the same one?**     **(Dependent on stage of education, if prior to school then we will have to go back to early years and when the need for additional support was first noticed)**     1. **How old was (name) when they first went to school?**      1. **Did (name) stay at this primary school up until secondary school?**  - **(If no) where did they move to? Was this the only move?**      1. **Did (name) have anything that they particularly liked about school then, or that they were good at? (e.g., favourite subject, extra-curricular activity)**      1. **Did they have anything that they didn’t like or found particularly difficult?**      1. **Do you remember how old (name) was when someone first spoke to you about their need for extra support?** 2. **Who was this person?** 3. **How did the conversation come about?** 4. **Can you remember when (name) first say they found some things harder to do?**      1. **Do you remember the first support (name) received?** 2. **What was it?** 3. **How was it recorded?** 4. **Was it monitored/updated?** 5. **Was it helpful?** 6. **Did this support have an impact on their health?**      1. **Did this support change over time?**  - **(Key transition points)**      1. **As a parent were/have you been included in the process?** 2. **Were you asked what would help?** 3. **Did the teachers speak to you?**      - **You know our research project is called HOPE – this stands for Health Outcomes for Young People in Education**      1. **I’d be really interested in whether you think the SEN support your child has received over the years has made a difference to their health – physically – mentally?** 2. **Has this changed over time?** 3. **What might have happened without it?** 4. **What might have happened with more appropriate provision?** | ***Address research questions:***     1. ***What are the effects of SEN provision on the Health Outcomes of Children and Young People with SEN?***      1. ***Is SEN provision fair and equitable across the country?***     ***Key areas to cover:***    ***When did you first think that they may have needed some extra support?***    ***Explore any previous SEN intervention/support they received?***   - ***What/Any others?*** - ***When/Timely?*** - ***Why/Does it match need?*** - ***Who/Services/ How was it decided?*** - ***What is working/enablers*** - ***How could it have been better/barriers*** - ***Made a difference/How?*** - ***Has this changed over time***     ***Key moments / transitions over education trajectory…***  ***(Transition to school/secondary school/GCSE/A-Levels/Higher education)***    ***Did this support have an impact on their health?*** |
| **The future – Support and Aspirations**     - **Thinking about the next few years …. Education … future**      - **Would you like to choose a different colour for the future or stay with the same one?**      1. **What would you like (name) to be doing in five years?**  - **In terms of education**      1. **Do you think their support will need to change to help with any future education/moving into adulthood?**  - **How would you like it to change?** - **Do you think they would like it to change?** - **How would you like to be involved in that decision-making?** - **What impact would this have on their education/health?** - **If things don’t change what impact would this have?**      1. **What do you imagine (name) being / doing in 5 years’ time when they’re XXX years old?**  - **What does (word) look like to you** - **What might help?** - **What might get in the way?**     **Anything else to add?** | ***Address research questions:***   1. ***What are the effects of SEN provision on the Health Outcomes of Children and Young People with SEN?***      1. ***Is SEN provision fair and equitable across the country?***     ***Key areas to cover:***    ***Need for SEN intervention / support for future?***   - ***What/Any others?*** - ***When/Timely?*** - ***Why/Does it match need?*** - ***Who/Services/ How should it be decided?*** - ***What is working/enablers*** - ***How could it be better/barriers*** - ***Make a difference/How?***     ***Would this support have an impact on their health?***    ***Anything else to add?***    ***Information about follow up:***   - ***Debrief email/UPS5 form/Copy of timeline*** - ***Withdrawal from study*** - ***Contact us by email with any questions***   ***Thank you*** |

## 3.0 Final coding framework

| **No.** | **Theme/**  **Subtheme** | **Definition and examples** |
| --- | --- | --- |
| **1.0** | **Child characteristics and participation in design of provision** | |
| 1.1 | SEND-related characteristics | SEND-related characteristics (e.g. some autistic traits) that can hinder asking for support, make it harder to engage with non-tailored ‘ordinarily available’ support, or that can be more difficult to identify / diagnose and determine support needs, or where the SEND label creates stigma or prejudiced attitudes (e.g. ‘you don’t really have autism’) which in turn is a barrier to being offered or asking for extra help. |
| 1.2 | Child voice and agency | To what extent the child has been asked or been able to contribute to the design of their support or speak up about how it’s going. |
| **2.0** | **Parent characteristics and family environment** | |
| 2.1 | Understanding, advocacy and engagement | Ability to advocate and “battle” for their child, parent engagement with other services (e.g. takes child to lots of playgroups, which speeds up identification, and to cadets which helps with self-esteem and behaviour in class), and to find new pathways to help their child (e.g. COVID pathway opened up more opportunities for some children). |
| 2.2 | Family environment | Complexity of home life, separation, visitation. |
| 2.3 | Home-schooling capacity | Parent as teacher and manager of personal budget, and employing therapists and teaching assistants. |
| **3.0** | **Early years, school/teacher and higher education characteristics** | |
| 3.1 | Teacher and senior leaders’ understanding about SEN & knowledge of child | **People included:** nursery and school teachers, teaching assistants, tutors, lecturers, head teacher, SENCO, school nurse  Coding examples: misunderstanding of SEND (e.g. how it presents, specific triggers), variable implementation of reasonable adjustments, whether educational aspirations for children are appropriate or too low, tailored and flexible teaching styles (some teachers can ‘change everything’, supply teachers can be problematic, inconsistency from year to year, teacher to teacher). Gap in support & unmet need, missed opportunities.  Senior staff permission to allow flexibility of school curriculum e.g. to drop foreign language due to language problems, supportive/initiative about assessments and to push for SEND funding, to prepare and manage transitions between school years (loss of information between years 7 & 8), thresholds for providing support not always appropriate – should be considered against child’s potential (physical and educational) not only triggered when grades are below average. Good documentation to facilitate transitions, positive reports about early identification in early years/nursery. School wider ethos around SEND, gaps in educational support, unmet need and missed opportunities. Long periods where child is out of education, nowhere to go, gaps in post-16 support. |
| 3.2 | Quality and appropriateness of communication and relationship with child and parent/carer | Aspects of communication about support, e.g. about why effective provision was removed, emails unanswered, unclear how the child is being monitored/reviewed, extent parent is consulted about decisions and how this affects trust and understanding what is happening at school.  Flexibility of communication – different options for communication for different parents. |
| 3.3 | School environmental factors | e.g. small class sizes, small group learning, extra-curricular and lunchtime offers for children (chess club, access to the library), zero-tolerance approaches and behavioural management strategies. Moving settings and other transitions. Influence of COVID-19. |
| **4.0** | **Wider factors** | **Factors beyond the school setting that influence SEND provision** |
| 4.1 | Health sector and professionals | **People included:** General Practitioners, Child and Adolescent Mental Health Services (CAMHS), community paediatricians, nurses (excluding school nurse – see 3.2), educational psychologists  Coding examples: waiting lists (e.g. Child and Adolescent Mental Health Services), quality of transition planning (especially post-16 and post 18), fragmented services, appropriateness of communication to others, quality of meeting minutes/not matching verbal discussion, extent of follow up, quality of relationships and support with processes e.g. annual reviews, and advocacy role of health professionals , lack of advice & support. |
| 4.2 | Social care ++ | **People included:** Social workers, Child in Need services, autism inclusion worker, family worker, police, Children and Family Court Advisory and Support Services, judges, Disabled Student Allowance/student loans. Code as for other sectors. E.g. lack of advice & support. |
| 4.3 | Third sector and community groups | **People included:** SENDIASS, charities, community groups including SEND provision, and informal support networks such as other parents/carers.  Can include these as part of wider SEND provision (e.g. if the group is part of a local authority’s Local Offer for SEND) or if it influences the effectiveness of a child’s SEND provision (e.g. cadets helped the child engage with ordinarily available provision), or if a charity provided support to a parent to help them secure provision for their child. e.g. benefits of sports (role models, social aspects, and expectations about behaviour) was transferred back into the classroom, making their educational experience better, and potentially their SEND-related support more effective. lack of advice & support. |
| 4.4 | LA-level factors | **People included:** SEND case workers, specialist teachers who do not meet the child but observe for a fixed number of sessions, LA-representatives on decision making panels.  Delayed provision worsened child’s readiness for transition, overwhelmed SEND teams, stuck between two LAs so no speech and language therapy provision, funding based decision making (rather than needs based), paperwork/bureaucracy/amount of evidence asking from schools is causing delays, difficult to convene ‘everyone in the room’, access to social security was good for one ppt, issues when change plans. Lack of advice & support. |
| 4.5 | National legislation and policy | e.g. SEND reform legislation leading to change in process and bureaucracy. The move from statements to EHCPs and how this affected diagnosis, identification, provision. Increasing/high cost of specialist places. Department for Education priorities. E.g. national curriculum and cursive writing don’t work well for some children with SEND, academisation negatively affected communication quality with some parents, ideological push to mainstream, teacher training on SEND only one day. |
| 4.6 | Interagency working and multi-agency decision making | Some parents discussed about nursery staff working with GP and parent in the stages leading up to diagnosis of autism others about not being listened to by multiple agencies. Blame culture, parent blaming and passing the buck. Opaque decision making/unlawful and disrespectful/disparaging to and about parents in Subject Access Requests. When a flag is raised about a child, but is not followed up or is rejected/told “there is nothing wrong with him”, then cycles around between nursery, GP, paediatrician who says nothing is wrong as well. Being stuck in a loop with professionals (who are perhaps not listening), and they have fallen through the system. |
| **5.0** | **Outcomes following SEN provision** (Child, family + wider e.g. societal) | |
| 5.1 | Child – Educational | Punishment (negative behaviour ‘points’), detention, exclusion, school attendance/refusal, attainment (e.g. reading level improved vs lost access to classes he enjoyed because taken out for SEN support, secondary school setting (school said child did not have the inner ‘drive’ needed to go to a grammar), positive ‘learning experience’/enjoyment. Off-rolling. |
| 5.2 | Child – Social, emotional, physical and mental health and wellbeing | Positive outlook and motivation, loneliness (due to unmet need?),poorer mental health, sleep/rest, school-based trauma, social outcomes e.g. feeling isolated –as long as it is related to SEND provision or a gap in provision that means they are stuck at home away from friends etc. |
| 5.3 | Child – Independence beyond school and into adulthood | How adequately does SEND provision prepare children for adulthood as independently as possible? Readiness for adult living/independence. Beyond school & preparation for adulthood and independence. Transition into adult services, social work, and employment dealing with additional vulnerability/increased risk of being exploited. |
| 5.4 | Parent/carer mental health, strain, worry, trust and satisfaction | Strain/stress experienced due to the SEND system or their child’s provision/lack of provision, leading some to give up securing SEND provision, dissonance -reverting to child’s teachers as the experts but unhappy with their decisions. Respite desperately needed for some.  Worries about adequacy of adult SEND provision – emotional stress and worry, and living with that, future cliff edge. Some parents do not fully trust the school, others were grateful. Delays / caught in a cycle / powerlessness / fallen through the system. |
| 5.5 | Family economic outcomes and opportunity costs | The costs of engaging with the SEND system or addressing children’s unmet needs for SEND themselves. e.g. Parents forced to give up job to home school |
| 5.6 | Expectations and aspirations for children with SEND as a determinant of outcomes | How SEND provision and in some cases the SEND label has either boosted or undermined / placed a ceiling on children’s future academic attainment and aspirations for adult life, or channelled them towards a particular educational route. How additional support threshold can be impersonal (e.g. below average grade, rather than below what that child could achieve). ‘Knowing the child’ and listening to the child and parent/carer about what they want and can achieve. Can be any point of view (child’s own, parent/carer, teacher). Discussion of cases where a child's unique strengths/gifts or potential is nurtured, enabling them to thrive. This perspective highlights the importance of recognizing a child's gifts, not just their difficulties, in the context of SEND provision. Also includes societal outcomes/bigger wider attitudinal change. |

## 4.0 Deviant cases

- **#226** The parent/carer emphasised the importance of social support from family, friends and other people going through similar things: “luckily we've had close friends and family around us who have been supportive throughout this entire thing. I don't actually know if I would’ve got through it properly without the support that we did have, the support that we did have came from like I say everyday people, friends and families, other parents and carers.”
- **#211** had overwhelmingly positive and consistent experience to date. because she's looked after to be honest with you, because she's a looked after child, they have a statute of responsibility to, to make sure that she's well, looked after, safe. And also that we are as well“.
- **#247**: Sometimes interagency working resolved issues for parents: “We spoke to the school and we asked our physio to assess and make a visit or and, and our OT actually so physio and OT did a school inspection and made their recommendations on what [CYP NAME] needed. “ and the EHCP helped to reinforce it: “they were, what’s the word, reluctant to allow her access to technology, and so that in the EHCP has helped “…” And the provision of a quiet space for when she's overwhelmed, the provision of that social, emotional and mental health, sort of those classes to help her. So all of those things, I think, put together have helped.”. OT and physio worked together.
- Children and Family Court Advisory and Support Service (CAFCASS), forensic Child and Adolescent Mental Health Services (CAMHS), and police only reported in one case **(#201).**

## 5.0 COREQ checklist

**COREQ (COnsolidated criteria for REporting Qualitative research) Checklist**

A checklist of items that should be included in reports of qualitative research. You must report the page number in your manuscript where you consider each of the items listed in this checklist. If you have not included this information, either revise your manuscript accordingly before submitting or note N/A.

| **Topic** | **Item No.** | **Guide Questions/Description** | **Reported on Page No.** |
| --- | --- | --- | --- |
| **Domain 1: Research team and reflexivity** |  |  |  |
| *Personal characteristics* |  |  |  |
| Interviewer/facilitator | 1 | Which author/s conducted the interview or focus group? | 8-9 |
| Credentials | 2 | What were the researcher’s credentials? E.g. PhD, MD | 8-9 |
| Occupation | 3 | What was their occupation at the time of the study? | 8-9 |
| Gender | 4 | Was the researcher male or female? | 8-9 |
| Experience and training | 5 | What experience or training did the researcher have? | 8-9 |
| *Relationship with participants* |  |  |  |
| Relationship established | 6 | Was a relationship established prior to study commencement? | 9 |
| Participant knowledge of the interviewer | 7 | What did the participants know about the researcher? e.g. personal goals, reasons for doing the research | 9 |
| Interviewer characteristics | 8 | What characteristics were reported about the inter viewer/facilitator? e.g. Bias, assumptions, reasons and interests in the research topic | 8-9 |
| **Domain 2: Study design** |  |  |  |
| *Theoretical framework* |  |  |  |
| Methodological orientation and Theory | 9 | What methodological orientation was stated to underpin the study? e.g.  grounded theory, discourse analysis, ethnography, phenomenology, content analysis | 8 |
| *Participant selection* |  |  |  |
| Sampling | 10 | How were participants selected? e.g. purposive, convenience, consecutive, snowball | 9 |
| Method of approach | 11 | How were participants approached? e.g. face-to-face, telephone, mail, email | 9 & Suppl. File |
| Sample size | 12 | How many participants were in the study? | 9 |
| Non-participation | 13 | How many people refused to participate or dropped out? Reasons? | Suppl. File, p.2 |
| *Setting* |  |  |  |
| Setting of data collection | 14 | Where was the data collected? e.g. home, clinic, workplace | 11 |
| Presence of nonparticipants | 15 | Was anyone else present besides the participants and researchers? | 10 |
| Description of sample | 16 | What are the important characteristics of the sample? e.g. demographic data, date | 10-12 |
| *Data collection* |  |  |  |
| Interview guide | 17 | Were questions, prompts, guides provided by the authors? Was it pilot tested? | 8 |
| Repeat interviews | 18 | Were repeat inter views carried out? If yes, how many? | 10 |
| Audio/visual recording | 19 | Did the research use audio or visual recording to collect the data? | 10 |
| Field notes | 20 | Were field notes made during and/or after the inter view or focus group? | 10 |
| Duration | 21 | What was the duration of the inter views or focus group? | 11 |
| Data saturation | 22 | Was data saturation discussed? | 9 |
| Transcripts returned | 23 | Were transcripts returned to participants for comment and/or correction? | 10 |
| **Domain 3: analysis and findings** |  |  |  |
| *Data analysis* |  |  |  |
| Number of data coders | 24 | How many data coders coded the data? | 10 |
| Description of the coding tree | 25 | Did authors provide a description of the coding tree? | Suppl. File, p.8-12 |
| Derivation of themes | 26 | Were themes identified in advance or derived from the data? | 10 |
| Software | 27 | What software, if applicable, was used to manage the data? | 10 |
| Participant checking | 28 | Did participants provide feedback on the findings? | 10 |
| *Reporting* |  |  |  |
| Quotations presented | 29 | Were participant quotations presented to illustrate the themes/findings?  Was each quotation identified? e.g. participant number | Results section |
| Data and findings consistent | 30 | Was there consistency between the data presented and the findings? | Results section |
| Clarity of major themes | 31 | Were major themes clearly presented in the findings? | 12 |
| Clarity of minor themes | 32 | Is there a description of diverse cases or discussion of minor themes? | Suppl. File, p.13 |

Developed from: Tong A, Sainsbury P, Craig J. Consolidated criteria for reporting qualitative research (COREQ): a 32-item checklist for interviews and focus groups. *International Journal for Quality in Health Care*. 2007. Volume 19, Number 6: pp. 349 – 357
